# Oral supplementation with ginseng polysaccharide promotes food intake in mice

**DOI:** 10.1002/brb3.1340

**Published:** 2019-08-08

**Authors:** Jiawen Wang, Yongxiang Li, Pei Luo, Yuhuang Chen, Qianyun Xi, Hanyu Wu, Weijie Zhao, Gang Shu, Songbo Wang, Ping Gao, Xiaotong Zhu, Yongliang Zhang, Qingyan Jiang, Lina Wang

**Affiliations:** ^1^ Guangdong Provincial Key Laboratory of Animal Nutrition Control, College of Animal Science South China Agricultural University Guangzhou People’s Republic of China; ^2^ National Engineering Research Center for the Breeding Swine Industry, South China Agricultural University Guangzhou Guangdong People’s Republic of China

**Keywords:** blood glucose, feeding behavior, hypothalamus, panax, polysaccharides

## Abstract

**Introduction:**

Ginseng polysaccharide (GPS, same as Panax polysaccharide) is a kind of polysaccharide extracted from ginseng. It has been reported that GPS has the ability to activate innate immunity, regulates blood sugar balance, and improves antioxidant capacity, but the effect on feeding behavior and its mechanism remains unclear.

**Method:**

To investigate the possible effect of GPS on feeding behavior of animals, mice were supplied with GPS in water, and food intake, hedonic feeding behavior, anxiety‐like behavior, expression of appetite‐regulation peptides in the central nervous system and glucose‐related hormone levels in the serum of mice were measured.

**Results:**

Ginseng polysaccharide significantly increased the average daily food intake in mice and promoted hedonic eating behavior. Meanwhile, the levels of serum glucose and glucagon were significantly reduced by GPS, and GPS promoted hypothalamic neuropeptide Y expression, inhibited proopiomelanocortin (POMC) expression, and reduced dopamine D1 receptor (DRD1) levels in the midbrain. We also found that the anxiety level of mice was significantly lower after GPS intake. In conclusion, oral supplementation with GPS promoted food intake in mice, most likely through the regulation of circulating glucose levels.

## 1 INTRODUCTION

Polysaccharides are organic macromolecules widely distributed throughout an organism. It is not only an important molecule that constitutes a cell membrane but also an essential component for the production of a variety of endogenous factors. Numerous studies have shown that polysaccharides can affect the immune system, nervous system, and metabolic system of organisms thereby improving immunity, producing antidepressant effects, and regulating glycolipid metabolism (Akhter, Mumin, Lui, & Charpentier, 2018; Liu et al., 2015; Schepetkin, Faulkner, Nelson‐Overton, Wiley, & Quinn, 2005). Researchers have verified ginseng polysaccharide (GPS, same as Panax polysaccharide) for safety and immune efficacy in healthy volunteers aged 50–75 years (Cho, Son, & Kim, 2014). Studies have found that GPS also affected dopamine (DA) neurons and had an antidepressant effect (Wang et al., 2010). GPS also reduced blood glucose levels in diabetic mice (Sun et al., 2014), which indicated that GPS can affect animal glucose metabolism. However, the effects of GPS on animal feeding have not been reported.

Generally, the feeding behavior of animals is affected by the appetite‐regulating system of the hypothalamus and the midbrain DA reward system. In addition, emotions such as anxiety can also affect the feeding behavior of animals.

The hypothalamus is an important region of the central nervous system (CNS) that regulates feeding. There are two major types of neurons in the hypothalamus: anorexigenic proopiomelanocortin (POMC) neurons and orexigenic agouti gene‐related protein (AgRP) neurons (Krashes, Lowell, & Garfield, 2016). AgRP neurons can produce an appetite‐promoting neurotransmitter, neuropeptide Y (NPY), which can synergize with AgRP to promote animal feeding behavior (Mercer, Chee, & Colmers, 2011). The hypothalamus can integrate peripheral signals such as hormones and adipose‐derived molecules with local signals to regulate the feeding behavior of animals (Tzameli, 2013). AgRP and POMC neurons expressing insulin receptors (IRs) are sensitive to central insulin actions and contribute to the regulation of systemic glucose homeostasis (Chen, Balland, & Cowley, 2017). The midbrain DA reward circuit is superimposed on this system and may override the signal from the hypothalamus, leading to overeating or extreme anorexia (Palmiter, 2007).

The feeding behavior of animals is also regulated by the hedonic system (Sasaki, 2017). Hedonic eating is a kind of feeding behavior that is driven by the rewarding effect and not dominated by the need for metabolism, and highly palatable energy‐dense foods are more likely to promote hedonic eating behavior (Ziauddeen, Alonso‐Alonso, Hill, Kelley, & Khan, 2015). The release of DA from the ventral tegmental area (VTA) plays an important role in the rewarding effect. It has been reported that activation of opioid peptide receptors can promote feeding by palatability (Kelley, Baldo, Pratt, & Will, 2005), and later studies have found that opioid peptide agonists can increase the level of DA in nucleus accumbens (NAc), producing feelings of pleasure and reward (Saigusa, Aono, & Waddington, 2017). The DA reward system is also regulated by hormones and nutrients. Neurons in the midbrain VTA and NAc also express hormone‐related receptors such as IRs and leptin receptors (Bruijnzeel, Corrie, Rogers, & Yamada, 2011). Additionally, the effects of hormones and nutrients on the hypothalamus or brain stem can also indirectly regulate the midbrain DA reward system (Steinbusch, Labouebe, & Thorens, 2015).

In addition to the direct regulation of feeding behavior by the CNS, emotions also affect animal feeding behavior. As early as 1989, people discovered that anxiety caused obesity and bulimia. Some scholars considered that anxiety behavior affects the hypothalamic–pituitary–adrenal (HPA) axis (Ulrich‐Lai, Fulton, Wilson, Petrovich, & Rinaman, 2015). Epel, Lapidus, McEwen, and Brownell (2001) found that anxiety can lead to an increase in glucocorticoid levels and increase the animal's preference for sweet foods. Anxiety not only changes the body's hormone levels to regulate feeding behavior but is also associated with the DA system. Zarrindast and Khakpai (2015) believes that DA levels in the midbrain and DA receptors play an important role in the regulation of anxiety. The effects of anxiety on hormones and DA systems suggest that anxiety may not only regulate animals' feeding behavior but may also have an impact on animals' hedonic feeding behavior.

To investigate the possible pathway by which GPS affects the feeding behavior of animals, mice were supplied with GPS in water, and food intake, hedonic feeding behavior, anxiety‐like behavior, expression of appetite‐regulation peptides in the CNS, and glucose‐related hormone levels in the serum of mice were measured.

## 2 MATERIALS AND METHODS

### 2.1 Animals

All experimental protocols and methods were approved by the College of Animal Science, South China Agricultural University. All experiments were conducted in accordance with “The Instructive Notions with Respect to Caring for Laboratory Animals” issued by the Ministry of Science and Technology of the People's Republic of China.

Four‐week‐old male C57BL/6 mice were purchased from Guangdong Medical Laboratory Animal Center, housed individually in cages and maintained on a 12:12 hr light/dark cycle (lights on from 6 a.m. to 6 p.m.). The mice were fed standard chow for 3 days to allow the mice to adapt to the environment. The control group was provided pure water, and the treatment group was provided water containing 1.5 g/L GPS (20% purity, Namiao Biotechnology Co. Ltd, China). The dose of GPS was based on published articles (Sun et al., 2014; Zhou, Shi, Jiang, Zhou, & Xu, 2014). According to the literature, the effective dose of oral GPS was 50–200 mg kg^−1^ day^−1^. We chose the lower dose. The purity of our GPS was 20%, and the water consumption of mice weighted 25 g is about 4–5 ml/day. Drinking 5 ml of 1.5 g/L GPS water a day means daily GPS uptake is about 7.5 mg (containing 1.5 mg effective GPS), that means 60 mg kg^−1^ day^−1^. Studies have shown that intermittent feeding of highly palatable food can activate the reward system (Avena & Bocarsly, 2012). We gave high‐fat diet (HFD) pellets (crude protein 18%, crude fat 60%, and crude ash 8%) on Monday and standard chow pellets (crude protein 18%, crude fat 4%, and crude ash 8%) for the rest of the weeks, and this weekly cycle was repeated.

### 2.2 Open‐field test

The open‐field chamber (60 × 60 × 60 cm) was surrounded by a black plastic plate, and the bottom was a white plastic plate. We divided the bottom into a grid pattern of 25 equal‐sized squares, with the nine squares in the middle defined as the center area, and the remaining 16 squares considered the surrounding area. Each mouse was placed in the center of the chamber and allowed to freely move for 5 min. Activity in the box was measured by total distance traveled, average speed, center entry counts, the amount of time, and distance traveled in the center area (measured by Supermaze) (Wang, Li, Du, Shao, & Wang, 2015).

### 2.3 Conditioned place preference

The conditioned place preference (CPP) apparatus consisted of two equal‐sized chambers (10 cm × 30 cm × 30 cm) that were separated by a sliding door. The CPP chamber was surrounded by a black plastic plate, the bottom was a white plastic plate; one chamber floor has circular holes, and the other chamber floor has linear holes. On day 1, to allow habituation, mice were allowed to freely access the apparatus for 15 min, and mice with a total time on one side more than twice the other side were excluded. From days 2 to 4, we closed the door, placed the HFD in the chamber with the floor with round holes, and defined it as the HFD area, and placed standard chow in the other chamber and defined it as the Chow area; mice were placed in the HFD area and Chow area, each for 15 min. On day 5, we formally evaluated the CPPs by opening the door and allowing the mice to move freely in the apparatus for 15 min. The time the mice spent in the two chambers was recorded.

### 2.4 Sampling

**Figure 1:**
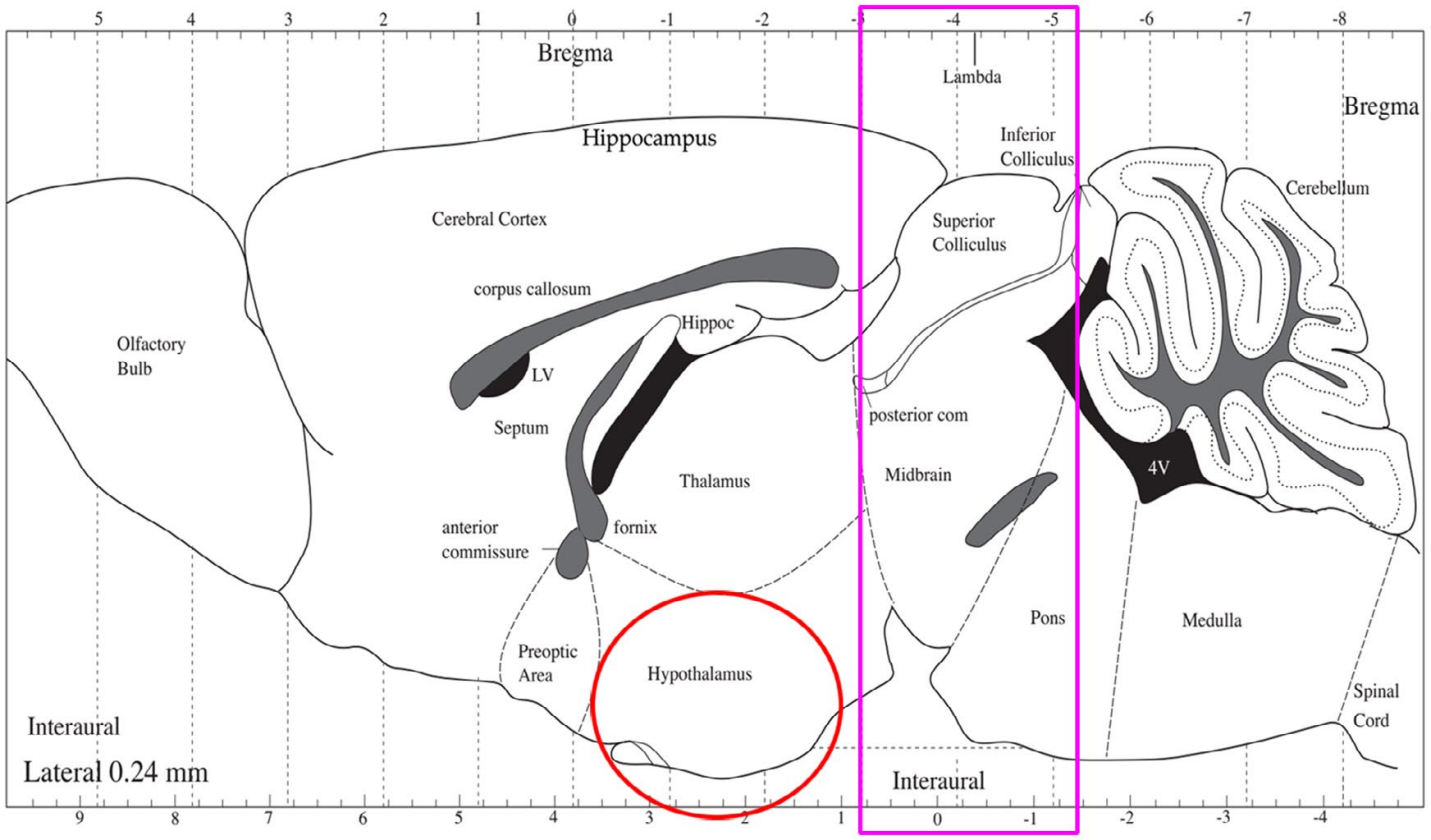
Sampling of the hypothalamus and midbrain in mice

Blood samples were collected from the orbital sinus and centrifuged at 4°C and 3,000 rpm for 15 min, and then serum was collected and stored at −80°C. After the completion of the blood collection, the mice were killed by cervical dislocation. POMC and AgRP neurons were mainly expressed in the arcuate nucleus at the bottom of the hypothalamus (Ilnytska & Argyropoulos, 2008; Young et al., 1998), so we extracted the brain and then removed the tissue at the bottom of the hypothalamus and stored it with liquid nitrogen. Then, in order to prevent brain deformation when separating the midbrain, we put the remaining brain into dry ice for a few minutes to shape, cut the midbrain along the pink line, and divide it into two halves along the midline of the brain (Figure 1). Samples were quickly frozen in liquid nitrogen and transferred to the −80°C refrigerator for preservation.

### 2.5 Glucose‐related metabolic indicators

Glucose in serum was detected by a glucose assay kit (F006, Jian Cheng, NanJing, China), and serum insulin and glucagon levels were detected by an ELISA kit (H203, H183, Jian Cheng, NanJing, China). Experimental steps were performed according to the instruction manual.

### 2.6 Polymerase chain reaction

The midbrain total RNA was extracted using TRIzol reagent (Invitrogen, Carlsbad, CA) according to the manufacturer's instructions. After treated with DNase I (2270A, Takara Bio, Kusatsu, Shiga, Japan), total RNA (2 μg) was reverse transcribed to cDNA in a final 20 μL by the M‐MLV Reverse Transcriptase (Promega, Madison, WI) and random 9 primer (Takara Bio Inc., Osaka, Japan) according to the manufacturer's instructions. SYBR Green Real‐time PCR Master Mix reagents (Toyobo Co., Ltd.) and sense and antisense primers (200 nM for each gene) were used for real‐time quantitative polymerase chain reaction (PCR). The results were normalized to the expression of the housekeeping gene β‐actin. PCR reactions were performed in an Mx3005p instrument (Stratagene, La Jolla, CA). The primer sequences are presented in Table 1 (Cai et al., 2016).

**Table 1:** PCR primer sequence of genes related to the dopamine reward system

Gene	Primer sequence (5′−3′)
DRD1	F: CAG TCC ATG CCA AGA ATT GCC AGA
	R: 5CCAAATCGATGCAGAATGGCTGGGTCT‐3C
DRD2	F: 5172AATCGATGCAGAATGGCTGGGTCT‐3T
	R: 5GATCTGGTGCTTGACAGCATCTC‐3G
DAT	F: 506‐AAATGCTCCGTGGGACCAATG‐3A
	R: 5GCTCGTCTCCCGCTCTTGAACCTC‐3C
β‐actin	F: 5′‐CCCTGTGCTGCTCACCGA‐3′
	R: 5′‐ACAGTGTGGGTGACCCCGTC‐3′

### 2.7 Western blot analysis

Radioimmunoprecipitation assay (RIPA) lysis buffer and protease inhibitor (Biosino Bio‐Technology and Science Inc., Beijing, China) were added to an appropriate amount of tissue according to the instructions. The tissue was ground in a homogenizer at a frequency of 60 Hz for 1 min. Finally, the lysed tissue was transferred to a centrifuge tube, centrifuged at 15294 *g* and 4°C for 10 min, the supernatant was separated, and the protein content was measured. The total protein of the tissue lysate was detected using BCA protein assays (Thermo Scientific Technologies, Wilmington, DE).

Equivalent amounts of protein (20 µg) were separated by 10% SDS‐PAGE, and the samples were transferred onto nitrocellulose membranes (Bio‐Rad, Hercules, CA). The samples were then blocked with 6% (w/v) nonfat dry milk in Tris‐buffered saline that contained Tween 20 for 2.5 hr at room temperature. The PVDF membranes were then subjected to immunoblotting with rabbit anti‐POMC (1:1,000, ab180766; Abcam), rabbit anti‐NPY (1:500, sc‐28943; Santa Cruz), rabbit anti‐β‐actin (1:2,000, bs‐0061R; Bioss), mouse anti‐DRD1 (1:1,000, sc‐33660; Santa Cruz), rabbit anti‐DRD2 (1:1,000, AB5084P; Millipore), and rabbit anti‐TH (1:1,000, AB152; Millipore). The primary antibodies were incubated at 4°C overnight, followed by incubation with the appropriate secondary antibody (Bioss) for 1 hr at room temperature. Western blots were visualized with SuperSignal West Pico Chemoluminescence substrate (Thermo Fisher Scientific) and quantified by ImageJ software.

## 3 RESULTS

### 3.1 GPS promoted food intake in mice

**Figure 2:**
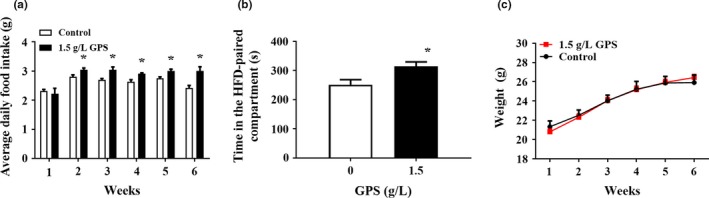
The effect of GPS on (a) average daily food intake, (b) CPP, and (c) weight of mice. Error bars indicate the *SEM*. *p < 0.05 versus mice in the control group

Supplementation of 1.5 g/L GPS in the drinking water significantly increased the average daily food intake of mice from the second week onward. Based on the fact that GPS promoted the food intake of mice, we investigated whether GPS affected the appetite of the mice. We used a CPP test to explore mice's preference for highly palatable food. Testing found that GPS increased the time the mice spent in the HFD region, indicating that GPS increased the hedonic feeding behavior of mice but had no significant effect on body weight (Figure 2).

### 3.2 GPS reduced anxiety‐like behavior of mice

**Figure 3:**
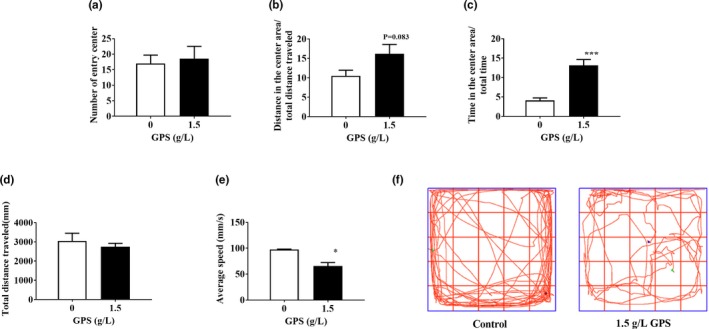
The effect of GPS on anxiety‐like behaviors of mice evaluated by the open‐field test. (a) The number of entries into the center of the open field. (b) Distance traveled in the center area relative to total distance. (c) Time in the center area relative to total time. (d) Total distance traveled in the open field. (e) Average speed in the open field. (f) Representative paths of mice in the open field. Error bars indicate the SEM. ^*^ *p* < 0.05 versus mice in the control group

To detect the effect of GPS on the anxiety‐like behavior of mice, we conducted an open‐field test. We placed the mice in the center of the open‐field chamber and allowed them to move freely for 15 min. The anxiety‐like behavior of the mice in each group during the open‐field session is summarized in Figure 3. The results showed that compared with the control group, the GPS group did not have an increased number of mice entering the center of the open field but the distance traveled and the time spent in the central area were increased. This showed that GPS relieved anxiety in mice. In the open field, we also observed that the average speed of the GPS‐treated mice was significantly reduced with the total distance unchanged. This shows that GPS reduced the autonomous activity of mice and had a calming effect.

### 3.3 GPS reduced blood glucose in mice

**Figure 4:**
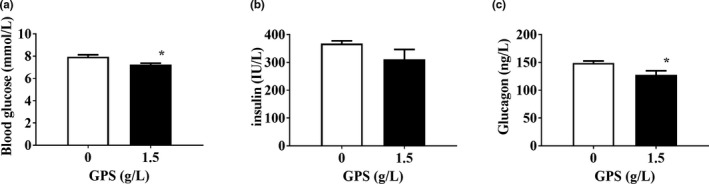
The effect of GPS on glucose metabolism in mice. (a) Blood glucose levels in mice detected by blood glucose test strips. (b) Serum insulin and (c) glucagon detected by an ELISA kit. Error bars indicate the SEM. *p < 0.05 versus mice in the control group

To investigate the effect of GPS on glucose metabolism in mice, we tested blood glucose‐related indicators in mice, and the results are shown in Figure 4. We found that GPS significantly reduced blood glucose and glucagon levels in mice but had no significant effect on insulin. We speculate that the effect of GPS on the feeding behavior of mice may be caused by affecting glucose metabolism.

### 3.4 Orexigenic effects in the hypothalamus and reward in the midbrain were activated by GPS

**Figure 5:**
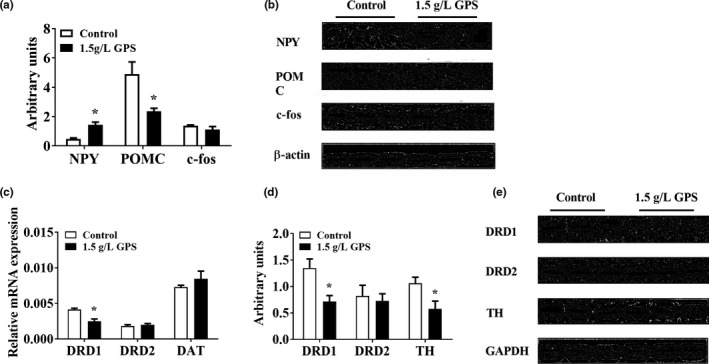
(a, b) The effect of GPS on the hypothalamus and midbrain. NPY, POMC, and c‐fos detected by Western blot. The effect of GPS on reward‐related RNA and protein in the midbrain. (c) DRD1, DRD2, and DAT detected by real‐time quantitative PCR. (d, e) DRD1, DRD2, and TH detected by Western blot. Error bars indicate the SEM. *p < 0.05 versus mice in the control group

To investigate the mechanism by which GPS promoted food intake, we examined the expression of appetite‐related proteins in the hypothalamus of mice. We found that GPS significantly increased orexigenic NPY expression and reduced anorexigenic POMC expression. There were no differences in the expression of c‐fos between the GPS‐treated mice and the control mice. To further explore the effect of GPS on the DA system in the midbrain, we examined the expression of DA receptors, DA transporter, and tyrosine hydroxylase (TH). We found that compared with the control group, GPS significantly reduced the expression of dopamine D1 receptor (DRD1) and TH but had no significant effect on the expression of dopamine D2 receptor (DRD2) and dopamine transporter (Figure 5).

## 4 DISCUSSION

Different polysaccharides have different effects on the feeding behavior of animals. A study by Wee showed that soy proteoglycan had no effect on animal energy intake (Wee, Yusoff, Chiang, & Xu, 2017). Gao et al. (2015) found that cactus polysaccharide significantly reduced the food intake and water intake of diabetic mice caused by streptozotocin, while weight gain was enhanced. Lentinus edodes polysaccharide alleviated abnormal feeding behavior caused by TNF (Tamura, Tanebe, Kawanishi, Torii, & Ono, 1997). A large number of studies have shown that polysaccharides may regulate animal metabolism and feeding. In our study, GPS significantly increased the average daily food intake and the preference for HFDs in mice probably through regulation of circulating glucose levels. But the weight of mice did not change significantly. We determined the body composition of the mice and found that the muscle ratio decreased significantly, while the thymus and the spleen indexes increased significantly (Figure S2). Studies have shown that GPS as an immune adjuvant can increase the weight of immune organs (Ilnytska & Argyropoulos, 2008; Kwast et al., 2016). In addition, studies have shown that GPS can promote energy metabolism in the body by increasing the ratio of ATP/ADP and ATP/AMP. Therefore, the energy intake may be used to improve immunity and metabolism (Li, Chen, Jin, & Chen, 2009).

When an animal's blood glucose concentration is lowered, feeding behavior is promoted. Some studies have shown that glucose can directly inhibit the secretion of AgRP in the hypothalamus (Chalmers, Jang, & Belsham, 2014). A large number of experiments have indicated that GPS has a significant effect on the sugar metabolism of animals. Xie, Wu, Mehendale, Aung, and Yuan (2004) found that an injection of GPS into obese mice significantly reduced their fasting blood glucose levels without affecting the body weight of the mice. Sun also found that GPS had hypoglycemic and antioxidant activities in diabetic mice (Sun et al., 2014). Our study found that GPS promoted animal feeding behavior and significantly reduced blood glucose and serum glucagon levels in mice. It is speculated that GPS may regulate the expression of appetite‐associated proteins in the hypothalamus by lowering blood glucose and serum glucagon levels.

Activating the midbrain DA reward system can make an animal feel pleasant, and a positive strengthening effect can promote hedonic feeding behavior because DA binds to its receptor and activates downstream signaling pathways to generate reward effects (Marsden, 2006). A Watanabe study found that added ginseng extract significantly increased striatal DA utilization in young mice and reduced autonomic activity in mice in the fifth week (Watanabe et al., 1991). The Sclafani study showed that a small amount of polysaccharide was more palatable than maltose, sucrose, and glucose, and the polysaccharide activated the reward system of mice (Sclafani & Clyne, 1987). We found that in the CPP test, GPS significantly increased the preference of mice for the HFD, and the level of autonomous exercise in the GPS‐treated group was significantly reduced in the open‐field test. These findings all suggest that GPS increased the utilization of DA in mice and increased their preference for palatable foods. Many researchers have found that DA has different effects on rewards, which may be due to the different subgroups of DA (Lammel, Lim, & Malenka, 2014).

Based on structural and pharmacological functions, five different DA receptors have been divided into two broad categories: the D1‐like receptors, which stimulate intracellular cAMP levels, D2‐like receptors can inhibit intracellular cAMP levels (Zhou & Palmiter, 1995). Dopamine receptors may be associated with a positive strengthening effect in the DA reward system. D1 receptor antagonists can block the expression of cocaine‐CCP in mice (Galaj, Manuszak, Arastehmanesh, & Ranaldi, 2014). More studies have shown that various drug rewards and natural rewards can be attenuated by DRD1 antagonists and D2 receptor antagonists (Elmer et al., 2005). We found that the expression of D1 receptors was decreased in the GPS‐treated group. Studies have shown that activation of the D1 receptor can inhibit eating of mice (Prado & Luis‐Islas, 2016). Durst found that intrauterine protein‐restricted mice were prone to hyperphagia, and the expression of D1 receptor in the NAc was significantly reduced (Durst, Konczol, Balazsa, Eyre, & Toth, 2018). Therefore, the increase in food intake in mice may be associated with a decrease in D1 receptor levels.

The VTA, NAc, and amygdala are brain regions associated with reward effects, and the amygdala plays a fundamental role in the regulation of anxiety‐related behavior (Tye et al., 2011). It has been found that eating highly palatable foods has a rewarding effect, and withdrawal from these foods can cause a decrease in the ability to experience pleasure in animals and further cause anxiety. Studies have shown that in the reward circuit, the withdrawal from highly palatable foods can cause changes in DA and plasticity‐related signals, which may lead to anxiety and overeating (Sharma, Fernandes, & Fulton, 2013). Clinically, it also shows that anxiety is highly correlated with dopaminergic neurotransmission and DA‐related reward effects (Piazza & Le Moal, 1996). Lehner found that mice with low levels of anxiety had higher levels of DA in the brain than mice with high anxiety levels and were more sensitive to rewarding effects than mice with high anxiety levels (Lehner et al., 2014). These studies suggested that anxiety levels are associated with reward effects and that high levels of anxiety may reduce the reward effect. Our study found that GPS increased the preference of mice for HFD, and GPS reduced the anxiety level of mice in the open‐field test. Kim found that ginseng can produce anxiolytic effects by downregulating the expression of TH (Kim et al., 2010). We also found that the expression of TH in mice from the GPS‐treated group was significantly reduced. These data indicate that GPS may reduce the anxiety level of animals by reducing the expression of TH in the midbrain and increase the sensitivity to rewarding effects.

## 5 CONCLUSION

We found that supplementation with 1.5 g/L GPS in water can promote feeding in mice, mainly by increasing the average daily food intake and the preference for highly palatable food. This may be attributed to reduced peripheral blood glucose levels that regulate hypothalamic appetite‐regulating peptides while affecting the midbrain DA reward system. Moreover, GPS decreased anxiety in mice, which may also change the feeding behavior through the HPA axis.

## Data Availability

Data sharing is not applicable to this article as no new data were created or analyzed in this study.
