# Supplementary figures and images for: Oral supplementation with ginseng polysaccharide promotes food intake in mice
Source: Brain Behav. 2019 Aug 8;9(9):e01340. doi: 10.1002/brb3.1340 (PMC6749478; doi:10.1002/brb3.1340)

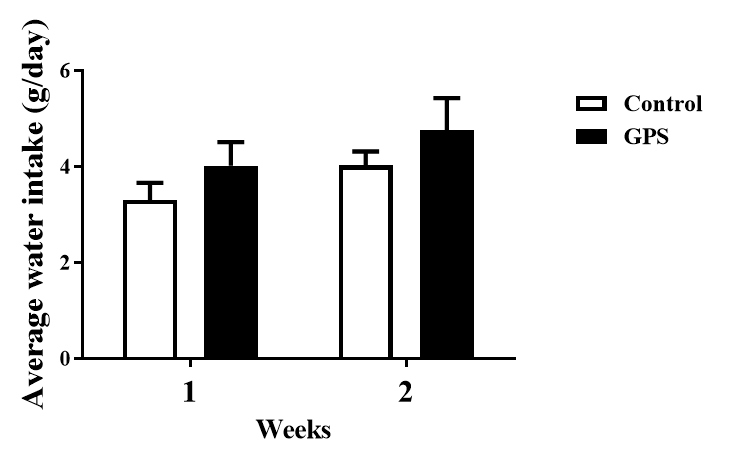

Supplement: Supplementary file 1 [file BRB3-9-e01340-s001.tif]

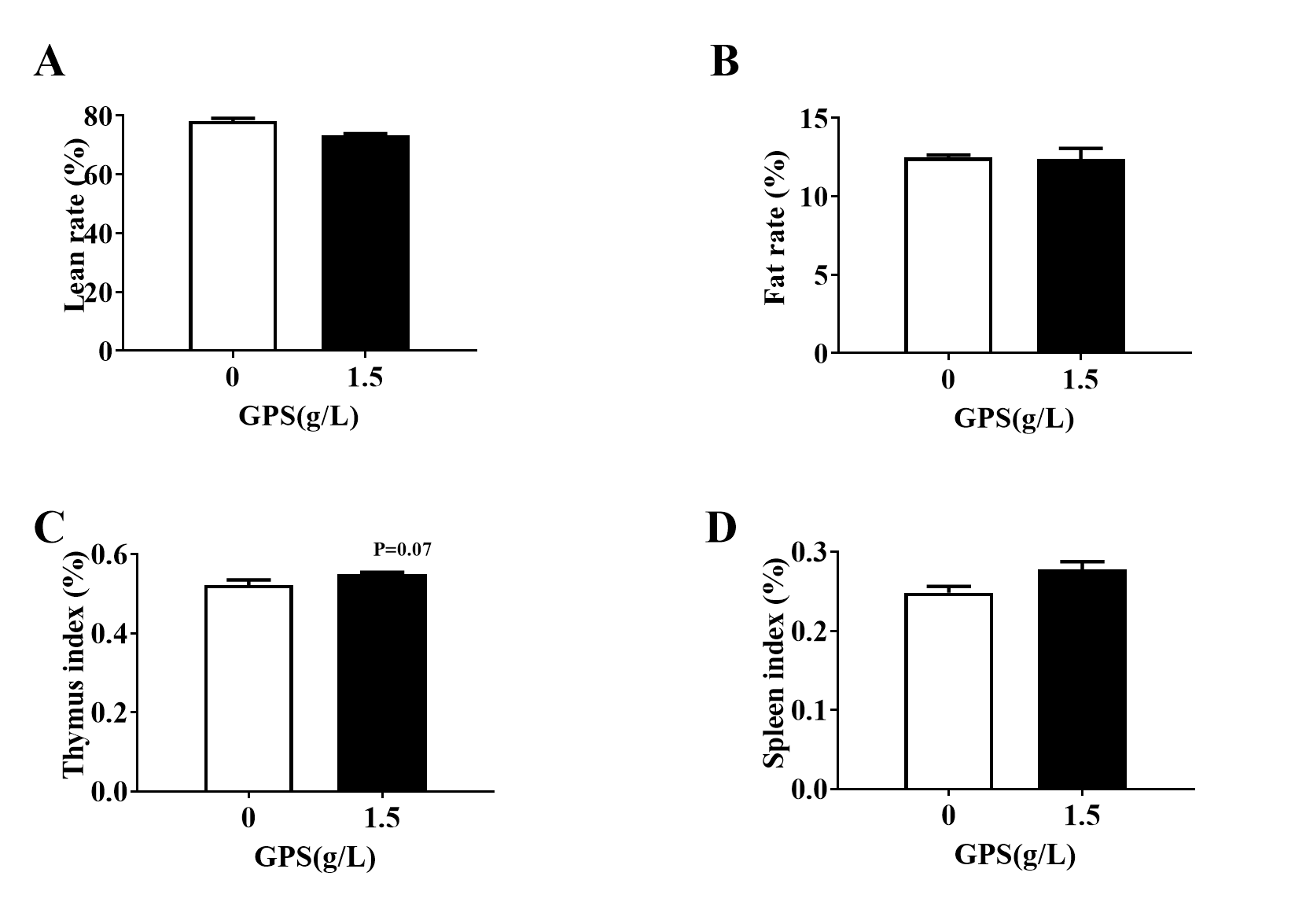

Supplement: Supplementary file 2 [file BRB3-9-e01340-s002.tif]
